# Storage reliability prediction of electromechanical components based on virtual manufacturing and testing

**DOI:** 10.1016/j.heliyon.2023.e20549

**Published:** 2023-09-29

**Authors:** Yigang Lin, Tao He, Haotong Zhu, Yichen Lin, Qiuying Chen

**Affiliations:** a College of Electrical and Electronic Engineering, Wenzhou University, Wenzhou, China; b School of Electrical Engineering, Xian Jiaotong University, Xian, China; c Beijing Aerospace Automatic Control Institute, Beijing, China; d Technology Institute of Wenzhou University in Yueqing, Wenzhou, China

**Keywords:** Electromechanical component, Storage reliability, Virtual manufacturing, Virtual testing, Wiener process

## Abstract

Electromechanical components (EMCs) such as relays and contactors have been used extensively in industrial and military areas. The storage reliability of these EMCs has a direct impact on the reliability of the system that contains them. However, during the design phase, it is difficult to predict the storage reliability of EMCs because of few failure rate data of parts, as well as limited testing time and budgets. To address these problems, a virtual Manufacturing and testing method is proposed in this paper, so as to simulate the storage degradation process of batch EMCs. By considering the influence of the quality screening process in the manufacturing process, as well as the unit-to-unit variability of EMCs on the storage degradation paths and the overall life distribution of batch products, the storage failure distribution function is obtained, based on Wiener process. At the same time, the distribution of the diffusion coefficient in the degradation model and the failure distribution model is quantified by introducing testing data of related products as priori information, so as to reflect the uncertainty of the storage degradation process of EMCs. A case study of an electromagnetic relay is carried out to illustrate the effectiveness of the proposed approach.

## 1 Introduction

With increasing research in reliability analysis, researchers are realizing that the storage reliability and working reliability of products are equally important [1,2]. In particular, products like automotive airbags [3], aerospace products [4], missiles [1,5] and other single-shot products are in storage for most of their lifetime. Their storage reliability is generally considered as the same as their general reliability. For such products, accurately predicting their storage reliability is a basic requirement for reasonably determining their storage life, and ensuring that they work reliably after their storage period comes to an end.

Influenced by the long-term continuous or intermittent exposure to environmental stresses such as temperature, humidity, vibration, etc., the internal materials and parts of EMCs will gradually degrade during the storage process, resulting in the degradation of their performance and reliability. This paper focuses on the prediction method of storage reliability of EMCs.

Reliability block diagram is a common reliability prediction method for a variety of products, but not for EMCs. Based on the failure rate of components provided by the manufacturers or the users, the reliability of equipment or a system can be predicted relatively accurately [6,7]. However, for EMCs, since most of the internal parts are non-standardized products and failure rate is not available, it is difficult to accurately predict their reliability using this traditional method.

Degradation test is an important technical method in the reliability research of EMCs. In recent years, scholars have conducted in-depth research in this area. A test system was developed in Ref. [8] to monitor the electrical contact behavior of EMCs, and the performance degradation laws and failure mechanisms of three widely used contact materials were effectively analyzed based on the system. Based on the accelerated degradation tests, the storage failure mechanism, storage life prediction and storage reliability evaluation of typical EMC (electromagnetic relay) were studied in Refs. [4,[9], [10], [11]], and meaningful results were achieved. However, product degradation resulting from storage environmental stress generally takes place in a relatively slow manner compared with its operating conditions. Therefore, although the testing-based storage reliability prediction can obtain effective results, it has to face longer testing time and higher testing cost. Especially for products in the design stage, multiple design cycles (include trial production, storage reliability verification and design modification) are needed, which will result in the consumption of substantial economic and time costs.

For these issues, the virtual prototyping technology will be an effective solution. At present, based on component degradation data, degradation simulations and reliability analyses on certain circuits are possible [12]. However, the simulations of EMCs are complicated because their working process involves the coupling of mechanical, electrical, magnetic and other physical fields [13,14]. This is particularly the case for the simulation of the degradation process of batch EMCs, since it faces a large number of model reconstruction problems, and requires high workload. The purpose of this paper is to provide an efficient and economical simulation-based virtual testing method to obtain simulation-based storage degradation data for the storage reliability prediction of batch EMCs.

With the simulated storage degradation data, the reliability of batch EMCs can be predicted in various method. As a classical reliability modeling method, Wiener process can successfully describe a uniform and gentle degradation process caused by the gradual accumulation of small random losses [15]. This process coincides with the storage degradation process of EMCs. Nevertheless, when modeling with Wiener process, researchers usually construct a unified likelihood function and estimate only one paired drift coefficient and diffusion coefficient through the degradation data of multiple samples [16,17]. The drift coefficient characterizes the expectation of multiple samples' degradation trends, while the diffusion coefficient is determined not only by the uncertainty of the degradation process, but also by the unit-to-unit degradation trends variability of samples. When products are good consistency in the degradation process, the accuracy of both the degradation model and the reliability assessed by this modeling method are acceptable. However, for products with large differences in individual degradation processes between batch samples such as EMCs [[18], [19], [20]], the only paired drift coefficient and diffusion coefficient cannot accurately describe the storage degradation process of batch EMCs. As a consequence, the accuracy of the reliability prediction can hardly be guaranteed.

In summary, the storage reliability prediction of EMCs is faced with the following problems: 1) lack of failure rate data for the bottom level parts; 2) time-consuming and cost-ineffective storage tests; 3) the unified paired drift coefficient and diffusion coefficient cannot accurately reflect the storage degradation process of batch EMCs. To address these problems, a new storage reliability prediction approach is proposed in this paper, based on virtual manufacturing and testing. Firstly, an overview of the approach is given in Section 2. Section 3 details the virtual manufacturing and testing-based storage degradation analysis method of EMCs. Based on the storage degradation data of virtual samples and Wiener process theory, the failure distribution model and the reliability model of batch products are presented for the storage reliability prediction of EMCs. Section 4 predicts the storage reliability of a certain type of aerospace electromagnetic relay. Section 5 discusses and analyzes the results of Section 4. Finally, the conclusions of this paper are given in Section 6.

## 2 Procedure of the proposed approach

**Fig. 1:**
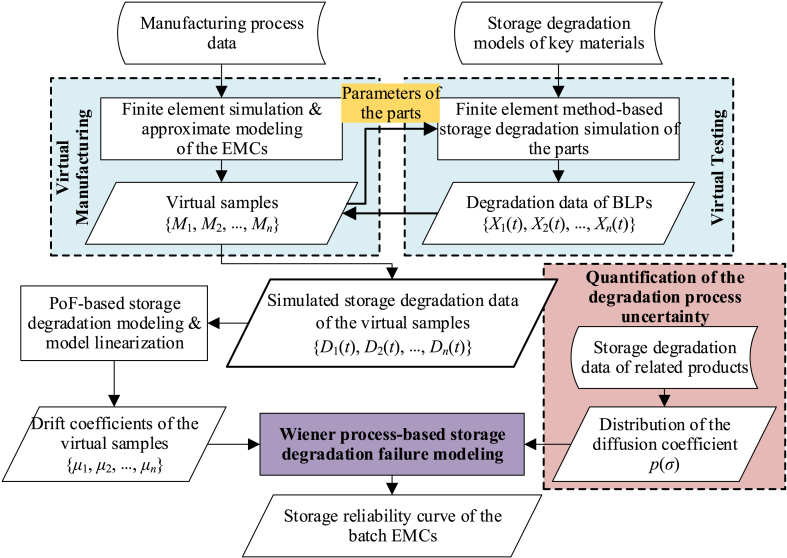
Flow chart of the proposed approach.

The core part of the proposed approach includes virtual manufacturing and testing for EMCs, as well as storage failure modeling and reliability prediction of batch products based on the acquired storage degradation data of virtual samples. The general procedure is shown in Fig. 1.

Firstly, based on the manufacturing process data of EMCs, the production process of batch EMCs is simulated by finite element method and approximate modeling techniques. The *n* virtual samples *M*_1_, *M*_2_, _…_, *M*_*n*_, which can fully reflect both the unit-to-unit variability and the statistical characteristics of batch products, are obtained to represent the virtual manufacturing of EMCs.

Failure mechanism analysis for EMCs have to be carried out, so as to identify the key materials that lead to the storage degradation of EMCs. At the same time, the storage degradation models of the performance of these key materials are obtained according to existing research results in materials science or through actual material-level storage degradation testing. According to the parameters of the virtual samples' parts obtained in virtual manufacturing, a finite element method is used to simulate the degradation process of these parts under the storage environmental stress *S*. Consequently, the simulated storage degradation data of the bottom level parameters (such as the size and property) of each virtual sample are obtained: *X*_1_(*t*), *X*_2_(*t*), _…_, *X*_*n*_(*t*). By directly adjusting the corresponding parameters in the finite element models or the approximate models, the data set can be then inserted into the corresponding virtual samples *M*_1_, *M*_2_, _…_, *M*_*n*_ in a time series. The storage degradation data of virtual samples corresponding to stress *S* are then obtained: *D*_1_(*t*), *D*_2_(*t*), _…_, *D*_*n*_(*t*). This completes the virtual testing of the batch EMCs.

Because of the ideal conditions of virtual testing, the simulated storage degradation paths of virtual samples are usually smooth curves. Therefore, these curves cannot reflect the uncertainty of the degradation process of EMCs. To address this problem, we model the storage degradation process of batch EMCs based on failure physics and Wiener process, to obtain the drift coefficients *μ*_1_, ‍*μ*_2_, ‍ _…_, ‍*μ*_*n*_ of *n* virtual samples. In addition, the storage degradation data of related products is introduced as a priori information to quantify the uncertainty of the storage degradation process of EMCs, and the probability density function (PDF) *p*(*σ*) of the diffusion coefficient can be obtained.

Finally, taking into account the effect of the manufacturing and screening process on the unit-to-unit variability of samples and the distribution characteristic of drift coefficients, a storage failure distribution model of batch EMCs reflecting the effect of the manufacturing and screening process is constructed. The corresponding storage reliability curve is calculated to achieve the storage reliability prediction of batch EMCs.

## 3 Storage reliability prediction of EMCs

### 3.1 Storage degradation analysis based on virtual manufacturing and testing

The finite element method can model and simulate the EMCs' action processes [13,14], so as to analyze the initial (the manufacturing has just finished, recorded as time of 0) output characteristic parameters *D*_*i*_(0) (*i* = 1,2, …,*k*) of the sample *i* whose parameter set is *P*_*i*_. In addition, the approximate modeling technique [21] is introduced to construct the fast calculation model *D* (0) = *G* (*P* (0)) of the output characteristics of batch EMCs, and generate *m* (*m* » *k*) virtual samples by combining the manufacturing process data. The calculation and analysis of both the output characteristics distribution and qualified rate of batch products can then be carried out. However, in engineering applications, the reliability of batch products that attract the general interest simply refers to the reliability of qualified products that have actually been delivered. Therefore, in order to accurately predict the storage reliability of batch EMCs in practical applications, it is necessary to simulate the quality screening of the manufacturing process. In this way, the unqualified products in the constructed virtual samples will be eliminate, and we can finally obtain *n* qualified virtual samples {*M*_1_,*M*_2_, …,*M*_*n*_} that can reflect the statistical characteristics of batch EMCs.

**Fig. 2:**
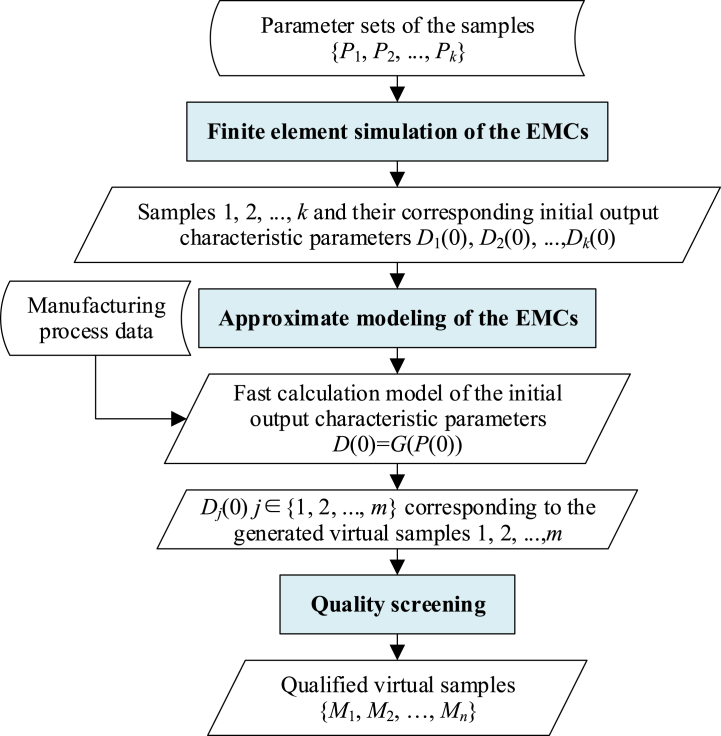
Virtual manufacturing of EMCs.

In this paper, the whole process (including the finite element simulation of individual, approximation modeling of batch samples and quality screening) is named the virtual manufacturing of EMCs. The basic process is shown in Fig. 2.

**Fig. 3:**
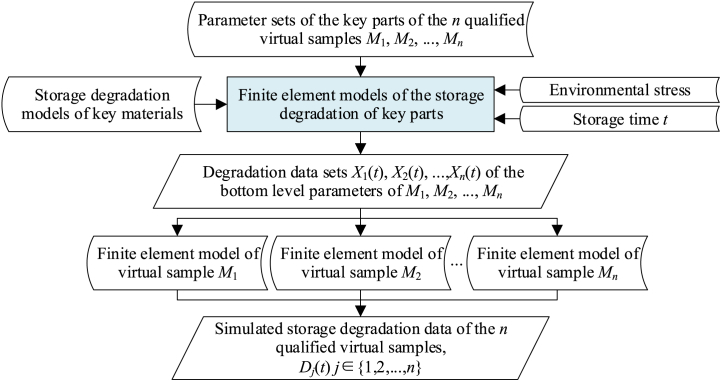
Virtual testing of EMCs.

However, the reality is that the storage reliability prediction for batch EMCs needs to be based on the degradation data of each sample. By simulating the storage degradation process of the parts in virtual samples, this paper therefore introduces the concept of virtual testing, to expand the output characteristic parameter *D*_*j*_(0) of any sample *j*∈{1,2, …,*n*} to a series of data points *D*_*j*_(*t*) which vary with the storage time *t*. The basic procedure is shown in Fig. 3.

Firstly, with *n* qualified virtual samples obtained from the virtual manufacturing, find out the key parts that lead to the degradation of EMCs, and construct the finite element models of the storage degradation of these key parts.

Secondly, insert the storage degradation model of the key materials, storage environmental stress and storage time *t* into these finite element models to simulate and analyze the storage degradation of the key parts of each virtual sample.

Next, denote *X*_*j*_ to be the subset of *P*_*j*_, in which the bottom level parameters will degrade during storage. Then *X*_*j*_(*t*) (corresponding to the storage time *t*) can be obtained, based on the results of storage degradation simulation of the key parts.

Finally, replace *X*_*j*_(0) in *P*_*j*_(0) with the corresponding *X*_*j*_(*t*) to get *P*_*j*_(*t*), and substitute *P*_*j*_(*t*) into the output characteristic fast calculation model constructed in the virtual manufacturing process. By this means, the virtual testing process is completed, and the storage degradation data (denote as *D*_*j*_(*t*) = *G* (*P*_*j*_(*t*))) of the virtual sample can be obtained.

Based on the acquired simulated storage degradation data, the storage reliability prediction of the batch products can be determined without relying on the tested storage degradation data of EMCs.

### 3.2 Wiener process-based storage reliability prediction

During the storage of EMCs, the continuous slow degradation of the inner materials will lead to many small losses in the output characteristics which will gradually accumulate. Thus, the storage degradation of the output characteristics of EMCs during the time interval Δ*t* can be regarded as a uniform and gentle degradation process caused by many small losses. The storage degradation process of EMCs can therefore be described by Wiener process.

According to the Wiener process, the expectation is that the degradation process is a linear function, and thus Wiener process is generally applicable to the modeling of products with linear degradation. However, the actual storage degradation process of many EMCs is often non-linear. A non-negative monotonically increasing function (*τ* = *Λ*(*t*)) of storage time *t* is needed, so as to conduct a time-scale transformation on the storage degradation process of EMCs according to their failure physics (thereby achieving the linearization).

Denote *D*_*j*_(*τ*) to be the value of the output characteristic of EMC sample *j* at time *t*, and the mathematical expression of the degradation amount Δ*D*_*j*_(*τ*) (equals to *D*_*j*_(*τ*) minus *D*_*j*_(Λ(0))) can be expressed as:

(1) $$\Delta D_{j}\left(\tau \right)=\mu_{j}\tau +\sigma_{j}W\left(\tau \right)$$

Where *j* is the number of the EMC sample, *μ*_*j*_ is the drift coefficient of sample *j*, *σ*_*j*_ is the diffusion coefficient, and *W*(*τ*) is the standard Brownian motion.

The PDF of *τ*_*j*_ corresponding to the storage life of sample *j* is given by:

(2) $$g\left(\tau_{j}|\mu_{j},{\sigma_{j}}^{2}\right)=\frac{\zeta }{\sqrt{2\pi {\sigma_{j}}^{2}{\tau_{j}}^{3}}}\exp\left[-\frac{{\left(\zeta -\mu_{j}\tau_{j}\right)}^{2}}{2{\sigma_{j}}^{2}\tau_{j}}\right]$$

where *ζ* represents the failure threshold.

For different sample individuals in batch EMCs, there are differences in their drift coefficients and diffusion coefficients due to the influence of manufacturing process, manufacturing error and storage environment uncertainty, etc. Therefore, the impact of these differences must be considered when predicting the storage reliability of batch EMCs. The independent variables *μ* and *σ* are used to represent the drift and diffusion coefficient of the individual, respectively. Denote their PDFs to be *l*(*μ*) and *p*(*σ*^2^), respectively. The PDF of *τ* corresponding to the storage life of the batch EMCs is then expressed as:

(3) $$f\left(\tau \right)=\int_{0}^{\infty }\int_{-\infty }^{\infty }g\left(\tau |\mu ,\sigma^{2}\right)l\left(\mu \right)p\left(\sigma^{2}\right)d\mu d\sigma^{2}$$

where the content of $g\left(\tau |\mu ,\sigma^{2}\right)$ is the same as that in Equation (2).

Storage life distribution estimation of batch EMCs requires exact expression of *l*(*μ*) and *p* (*σ*^2^). However, the drift coefficient *μ* is usually closely related to the unit-to-unit variability of products and the initial values of the output characteristics that characterize this variability. Due to the influence of product quality screening, the final distribution form and range of *μ* will usually be different from the theoretical distributions. In addition, since the impact of fluctuations in environmental factors on the product's storage degradation process is not considered in the virtual testing, the resulting storage degradation path is an ideal smooth curve. The curve obviously cannot fully reflect the uncertainty of the degradation process. To address this problem, this paper introduces historical testing data of related type of product as the priori information to quantify *p*(*σ*^2^).

Let $\hat{l}\left(\mu \right)$ and ($\mu_{U}$, $\mu_{L}$) to indicate the re-determined actual PDF of *μ* and its distribution range, respectively. Then, assume the corresponding *σ*_*j*_^2^ of every sample comes from the same inverse gamma distribution *p*(*σ*^2^) ∼ IGa(*α*, *β*). Equation (3) will be converted into:

(4) $$f\left(\tau \right)=\int_{0}^{\infty }\int_{\mu_{L}}^{\mu_{U}}g\left(\tau |\mu ,\sigma^{2}\right)\hat{l}\left(\mu \right)\frac{\beta^{\alpha }}{\Gamma \left(\alpha \right)}\sigma^{-2\left(\alpha +1\right)}\;\exp\left(-\beta \sigma^{-2}\right)d\mu d\sigma^{2}$$

where Γ(·) represents the Gamma function, and the distribution parameters (*α*, *β*) can be estimated from the historical testing data of the chosen related type of product.

**Fig. 4:**
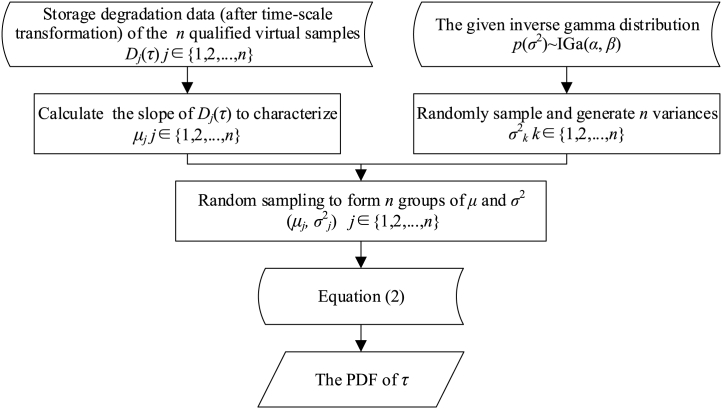
Numerical calculation flow of Equation (4).

Based on Equation (4), *F*(*τ*) can be estimated after integrating *f*(*τ*). However, for most cases, $\hat{l}\left(\mu \right)$ cannot be expressed by a specific distribution function. Thus, the integration of Equation (4) by analytical methods will always be impossible. Therefore, a numerical method as shown in Fig. 4 is proposed to approximate the integration process.

Based on the obtained PDF of *τ*, by inversely transforming *τ* = Λ(*t*) by time scale, both the storage failure cumulative distribution function *F*(*t*) and the corresponding storage reliability function *R*(*t*) of batch EMCs can be obtained.

## 4 Case study

**Fig. 5:**
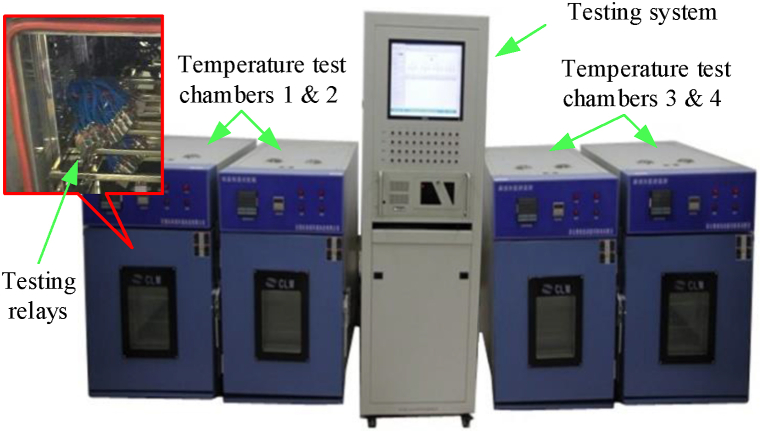
Self-developed storage degradation testing system and the actual testing samples.

As a typical electromechanical component, aerospace electromagnetic relays are widely used in long-term storage products such as missiles, rockets, etc. In this paper, an aerospace electromagnetic relay is chosen as an example application for the proposed storage reliability prediction approach. The effectiveness of the proposed approach is verified through comparing the reliability evaluation results of the virtual samples with those of 100 actual samples. As shown in Fig. 5, a self-developed testing system was used to perform the storage degradation testing for the actual samples under the storage condition of 170 °C. During the 7448 h of testing, the performance degradation processes of all 100 testing relays were continuously monitored.

Analysis of the relay structure and storage failure mechanism reveals that temperature stress is the key environmental stress that causes its storage degradation. Moreover, the stress relaxation behavior of the movable spring is the key factor leading to storage degradation of the spring force characteristics and the relay performance [22]. The external characterization of the stress relaxation behavior is the storage degradation of relay's release time. In this case, we assume that parts other than the movable spring of the relay are not subject to storage degradation, and that only the movable spring is used as a target for the virtual testing under the same storage condition as the actual samples. The release time is selected as the marker parameter for characterizing the storage degradation of the relay. The corresponding storage degradation data *T*_rls_(*t*) of the batch products will be obtained based on the virtual manufacturing and testing.

### 4.1 Virtual manufacturing of the relay

The unit-to-unit variability of the parts caused by tolerance and other factors in the manufacturing process are the main uncertainties affecting the storage reliability of batch EMCs. Table 1 shows the parameter fluctuation range of some parts in batch EMCs.

**Table 1:** Parameter fluctuation range of some parts.

Name	Armature height (mm)	Core radius (mm)	Yoke length (mm)	Coil resistance (Ω)	Preload of spring (N)
**Mean value**	0	0	0	0	0
**Standard deviation**	1 × 10^−5^	1 × 10^−5^	5 × 10^−6^	5	2 × 10^−2^

**Fig. 6:**
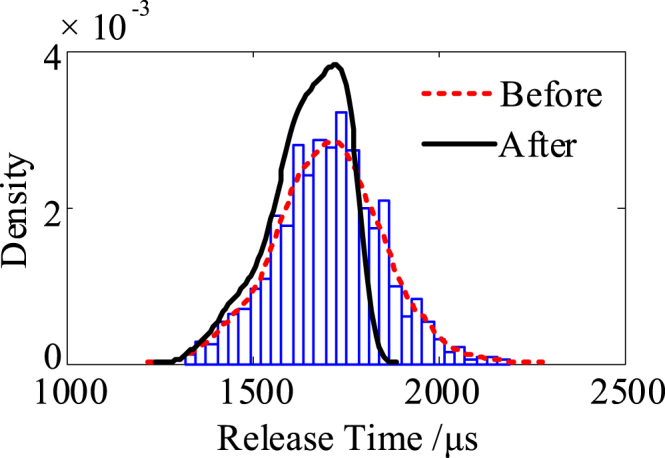
Distribution of virtual samples' release time before and after screening.

Based on the finite element simulation results, construct the surrogate model of the relay. Then, within the range of design parameters and manufacturing process data, 1000 virtual relays with different parameter sets are randomly sampled. After that, quality screening with 1800 μs as the upper limit is performed to complete the whole virtual manufacturing. The distribution of the release time before and after the screening is shown in Fig. 6. The red dotted line indicates the theoretical distribution of batch relays' release time, and the black solid line indicates the actual release time distribution of qualified relays (corresponding to the ones to be delivered). Further virtual testing will be conducted on qualified virtual relays that sampled from the demonstration range of black solid line, so as to reflect the statistical characteristics of actual batch relays.

### 4.2 Virtual testing of the relay

**Fig. 7:**
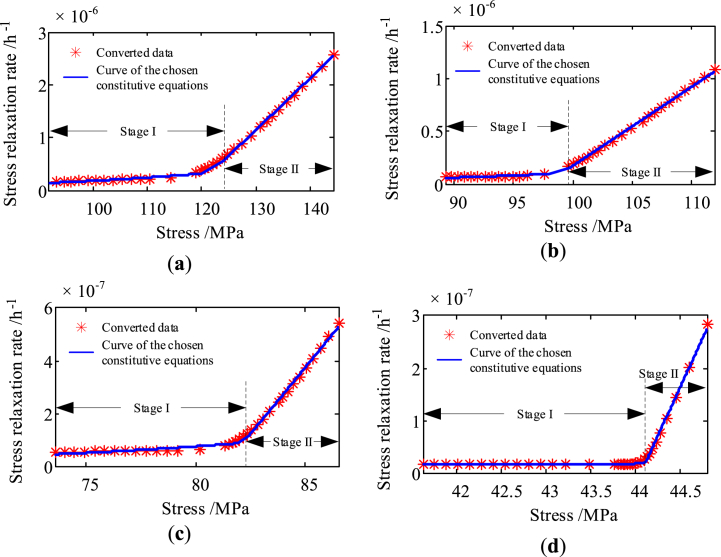
Stress relaxation rate-Stress data and the fitted curves. (**a**) Initial stress is 142.1 MPa; (**b**) Initial stress is 112.0 MPa; (**c**) Initial stress is 86.1 MPa; (**d**) Initial stress is 44.6 MPa.

100 samples are randomly chosen from the qualified virtual samples for virtual testing. In order to obtain the stress relaxation constitutive equations of movable spring, a storage degradation testing of AgMgNi 0.24–0.29 under four initial stress conditions is carried out at 170 °C. During the testing, the stress relaxation process has been continuously monitored and recorded. By transforming the tested data, the converted Stress relaxation rate-Stress data as shown by red * in Fig. 7 can be simply obtained.

As its shown in Fig. 7, the four curves can be divided into two different stages according to the rate of their change. In this paper, a piecewise function as shown in Equation (5) is chosen to be the stress relaxation constitutive equations, so as to describe the two stages of these curves. The corresponding model parameters are listed in Table 2, and the fitted curves are indicated by solid blue lines in Fig. 7.

(5) $$\left\{ \begin{array}{l} \dot{\varepsilon }\left(\sigma \right)=K\sigma^{n}\;\text{Stage}\;I\\ \dot{\varepsilon }\left(\sigma \right)=A_{1}\sigma +A_{0}\;\text{Stage}\;\text{II} \end{array}\right.$$

where $\dot{\varepsilon }\left(\sigma \right)$ is the stress relaxation rate corresponding to stress *σ*; *K*, *n*, *A*1, *A*0 are model parameters; the Stage I and Stage II are indicated in Fig. 7.

**Table 2:** Model parameters of the stress relaxation constitutive equations.

Initial stress (MPa)	*K*	*n*	*A*_1_	*A*_0_	Coefficient of determination
142.1	1.25 × 10^−13^	3.08	9.84 × 10^−8^	−1.17 × 10^−5^	0.9970
112.0	1.64 × 10^−18^	5.40	7.52 × 10^−8^	−7.35 × 10^−6^	0.9989
86.1	1.05 × 10^−19^	6.23	1.01 × 10^−7^	−8.22 × 10^−6^	0.9962
44.6	1.03 × 10^−12^	2.57	3.72 × 10^−7^	−1.64 × 10^−5^	0.9966

Based on the parameters of the movable springs of the chosen 100 relays, the finite element models of these springs are constructed. Combined with the obtained stress relaxation constitutive equations, the storage degradation process of each movable spring can be simulated, and the deformation data of these springs that vary with the storage time can be obtained.

**Fig. 8:**
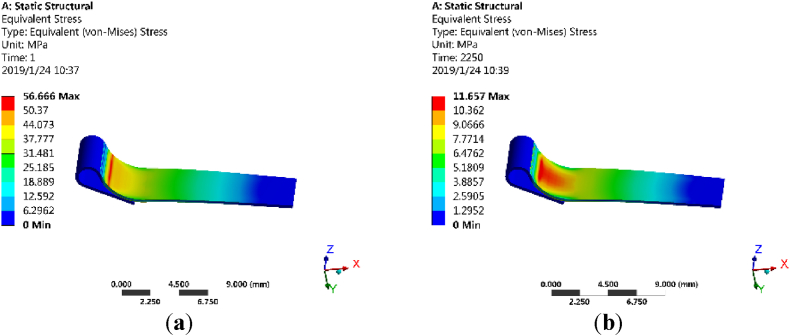
Stress distribution of the movable spring in release state. (**a**) Before the virtual testing; (**b**) After the virtual testing.

Before and after the virtual testing, the stress distribution of the movable spring in one of these virtual samples is shown in Fig. 8. As its shown, after virtual testing, the maximum stress on the movable spring is only about 20.57% of the initial state.

**Fig. 9:**
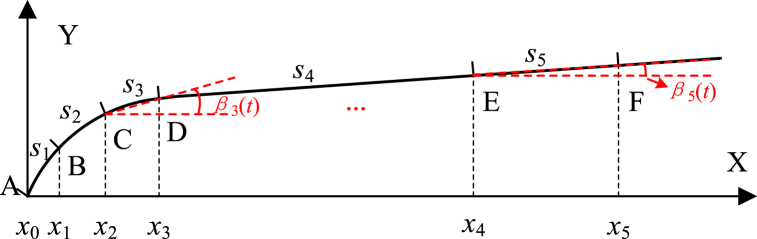
Equivalent structure and sub-flexibility segments of the movable spring at time *t*.

Based on the spring deformation data obtained from the virtual testing, virtual testing of the chosen samples can be implemented. As its shown in Fig. 9, the movable spring is divided into 5 sub-flexibility segments (*s*_1_,*s*_2_, …,*s*_5_), in order to construct the surrogate model with deformation energy method. Point *E* is the contact position of the armature push rod and the movable spring. Point *F* is the contact position of the movable spring and the stationary contact. *β*_*i*_(*t*) (*i* = 1,2, …,5) represents the angle between the flexibility segment *s*_*i*_ and the *X* axis at time *t*.

**Fig. 10:**
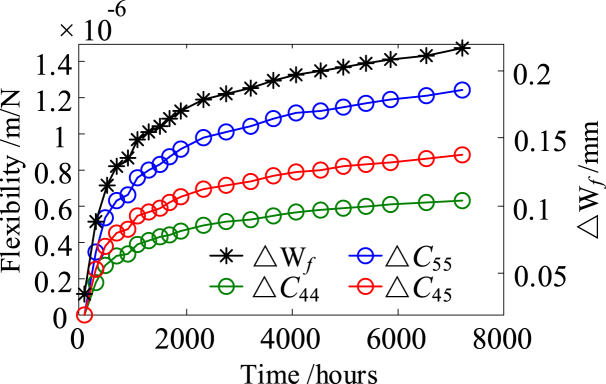
The changing flexibility of the movable spring and the deflection increment curve at point *F*.

Let ΔW_*f*_ represents the deflection increment of the movable spring at point *F*. Δ*C*_55_, Δ*C*_44_ and Δ*C*_45_ represent the amounts of change in the self-flexibility and the mutual-flexibility of point *F* and point *E*, respectively. The flexibility curves of the movable spring and the deflection increment curve at point *F* of a virtual sample during the virtual testing are shown in Fig. 10.

**Fig. 11:**
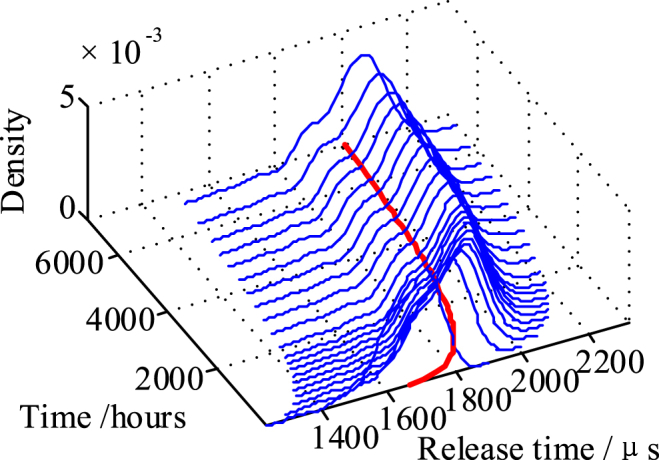
Simulated storage degradation distribution of sampled virtual samples.

The calculated flexibility of the movable spring and the deflection increment of point *F* of all qualified virtual samples are substituted into the constructed fast calculation model of the relay's dynamic characteristics. The release time storage degradation curves of the batch relays are obtained, with distribution characteristics shown in Fig. 11.

### 4.3 Storage degradation modeling and reliability prediction

Our previous research [18] demonstrates that the storage degradation process of the release time can be expressed as a function of its initial value *T*_rls_(0) and the Larson-Miller coefficient *θ*. To make the model meaningful at time *t* = 0, we replace the log*t* term in *θ* with log (*t*+1). The corresponding physics-of-failure-based model is shown in Equation (6).

(6) $$\left\{ \begin{array}{l} T_{\text{rls}}\left(t\right)=\left[T_{\text{rls}}\left(0\right)-c\right]\left(a\theta +b\right)+c\\ \theta =T\left[\log\left(t+1\right)+C\right] \end{array}\right.$$

where *T*_rls_(*t*) is the release time of the relay at storage time *t*; the unit of temperature *T* is Fahrenheit; *a*, *b* and *c* are model parameters; *C* is the material constant.

Let *τ* = Λ(*t*) = *T*log (*t*+1), Δ*T*_rls_(*τ*)‍ = ‍*T*_rls_(*τ*)‍-‍*T*_rls_ (Λ(0)), then the mathematical expression of Δ*T*_rls_(*τ*) can be obtained according to Equation (6).

(7) $$\Delta T_{\text{rls}}\left(\tau \right)=\left[T_{\text{rls}}\left(0\right)-c\right]a\tau$$

**Fig. 12:**
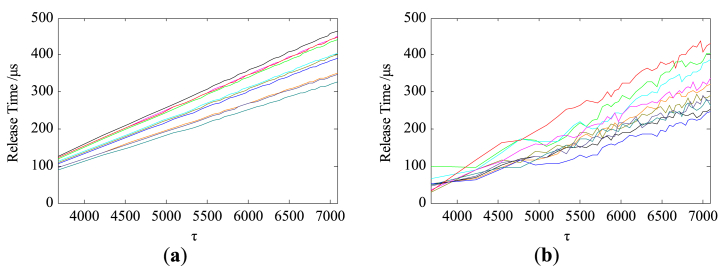
The time-scale transformed storage degradation curves. (**a**) Virtual samples; (**b**) Actual samples.

Fig. 12 shows the time-scale transformed release time degradation curves of part of the virtual relay samples as well as that of the actual samples.

The drift coefficient and the diffusion coefficient of the sample *j* are characterized by *μ*_*j*_ = *a*_*j*_[*T*_rls_*j*_(0)-*c*_*j*_] and *σ*_*j*_, respectively. *T*_rls_*j*_(0) is the initial value of the release time of the sample *j*. Then, according to Equation (1), the degradation model of the sample *j* with the Wiener process form is obtained.

According to the expression, *μ*_*j*_ is closely related to *T*_rls_*j*_(0). Since there is a set of unknown parameters (*a*_*j*_,*c*_*j*_), the samples with a same initial value are allowed to have different drift coefficients. This reflects the influence of the unit-to-unit variability on the samples' own degradation process.

For any qualified virtual sample *j*, the value of *μ*_*j*_ can be estimated according to *T*_rls_*j*_(*τ*). At this time, although *T*_rls_*j*_(0) is known, the expression *μ*_*j*_ = *a*_*j*_[*T*_rls_*j*_(0)-*c*_*j*_] is still an underdetermined equation, and the value of (*a*_*j*_,*c*_*j*_) cannot be accurately derived. This means that the exact expression of $\hat{l}\left(\mu \right)$ cannot be given, and the integral operation in Equation (4) cannot be performed. To this end, our approach directly calculates the drift coefficients of 100 qualified virtual samples based on the time-scale transformed release time, and obtains the drift coefficient set {*μ*_1_, *μ*_2_, …, *μ*_100_} of the batch qualified relays.

**Fig. 13:**
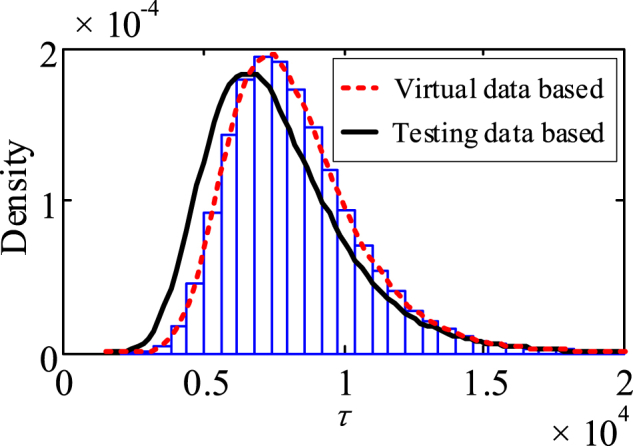
Storage failure distribution of the batch relays.

Based on the storage degradation data of a related type of product, *p*(*σ*^2^) ∼ IGa(13.6, 49.2) is obtained. After that, a corresponding set of diffusion coefficients {*σ*_1_, ‍ *σ*_2_, ‍ …, ‍*σ*_100_} is generated by randomly sampling from the (*σ*^2^). The drift coefficient set and the diffusion coefficient set are randomly combined, and the distribution of *τ* corresponding to each virtual sample can be obtained according to Equation (2). Assume that the failure threshold *ζ* is 700. By numerically simulating the distribution of *τ* corresponding to 100 qualified virtual samples, the overall distribution (as shown by the red dashed line in Fig. 13) is obtained based on the non-parametric model.

**Fig. 14:**
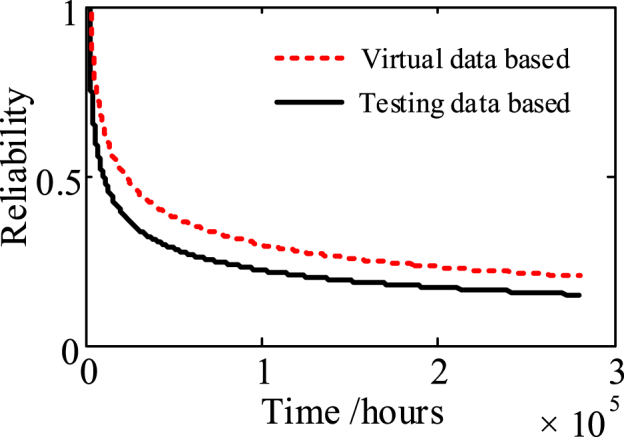
Comparison of the predicted storage reliability of the batch relays.

The time-scale inverse transformation is performed on *τ*, and the storage life of the relay is represented by *T*_*L*_. According to *R*(*t*) = *P*{*T*_*L*_ > exp (*τ/T*)-1}, the storage reliability of the batch relays based on virtual manufacturing and testing can be obtained, as shown by the red dotted line in Fig. 14.

To verify the effectiveness of the proposed approach, the storage degradation modeling and storage reliability evaluations are performed based on the storage testing data of 100 actual samples using Wiener process. The storage failure distribution curve and storage reliability curve of the actual samples are shown by the solid black lines in Fig. 13, Fig. 14, respectively.

The difference between the two reliability curves in Fig. 14 is characterized by mean absolute error (MAE) and mean absolute percentage error (MAPE), which are approximately 0.06 and 12.50%, respectively. In addition, the maximum absolute error and absolute percentage error between the curves are approximately 0.13 and 29.35%. These results show that the proposed approach could provide a relatively accurate prediction results of storage reliability of the studied relay.

## 5 Discussion

As shown in Fig. 13, Fig. 14, there are still differences between the predicted storage reliability obtained based on the two types of degradation data. These differences can be attributed to two things. Firstly, in the virtual manufacturing process this case has simplified and approximated the model to improve the calculating efficiency, resulting in some differences between the generated virtual relay samples and the actual samples. In addition, although the performance parameters of the parts and materials other than the movable spring (such as the coil, soft magnetic materials, etc.) have no obvious degradation tendency and exact degradation law during storage, the small fluctuations in them still inevitably affect the output characteristics and reliability of the relays. Nevertheless, so as to simplify the calculation of this case, such uncertainties have not been quantified in the virtual testing process. As a result, some differences between the storage degradation process of the virtual relay samples and that of the actual samples will be inevitable.

In summary, although the differences between the two reliability prediction results are objective, our results still indicate that the proposed approach is effective for predicting the storage reliability of the chosen aerospace electromagnetic relay.

It should be noted that the only key part that causes the storage degradation of the chosen relay is its movable spring. Based on the stress relaxation simulation method proposed in the case, the storage degradation process of the movable spring can be effectively simulated, thereby realizing the virtual testing of the relay. However, for other EMCs with more complex storage degradation causes, it is necessary to study the storage degradation simulation methods for more key parts (such as permanent magnet, etc.) before further predicting the storage reliability based on the virtual manufacturing and testing method proposed in this paper.

## 6 Conclusions and future work

### 6.1 Conclusions

In order to address problems in predicting storage reliability of EMCs, this paper proposed a new approach for simulating the storage degradation process of batch EMCs, as well as predicting their storage reliability. The main contributions are summarized as follows.

1) Based on combining the techniques of finite element simulation and approximate modeling with the parts' storage degradation simulation, virtual manufacturing and testing methods for EMCs were proposed. By using these methods, the storage degradation process of batch EMCs can be effectively simulated, and the problem that it's unable to efficiently obtain the storage degradation data of batch EMCs in their design stage can be addressed.
2) When modeling the storage degradation process based on Wiener process, by introducing the drift coefficient of distribution form as well as using the testing data of a certain related type of product as the priori information of the diffusion coefficient, it's possible to quantify the differences in the individuals' degradation trends, the effects of quality screening, and the uncertainty of the degradation process. By this means, the impact of manufacturing process, manufacturing error and the fluctuations of storage environment on the statistical characteristics of the storage degradation process of batch products can ultimately be reflected.
3) The results of a case study show that the storage reliability of the relays can be predicted based on the proposed approach without conducting any relay storage testing. The MAE and MAPE between the predicted storage reliability and the actual storage reliability are approximately 0.06 and 12.50%, respectively. The corresponding maximum absolute error and absolute percentage error are approximately 0.13 and 29.35%, respectively. The proposed approach provides a feasible solution to the problem that it is difficult to accurately predict the storage reliability of EMCs when there is a lack of failure rate data for their parts.
4) The approach proposed in this paper is suitable not only for predicting the storage reliability of EMCs in the design stage, but can also be a reference for predicting both the storage reliability and the operational reliability of various types of long-life products (whether component, equipment or system). In addition, the simulated storage degradation data obtained using virtual manufacturing and testing can also be used as priori information to address the problem of small samples in the reliability evaluation of products.

### 6.2 Future work

In this paper, the authors' main purpose is to propose a general approach of storage reliability prediction based on virtual manufacturing and testing, which can be applicable to different types of EMCs. However, more supplementary research is still needed to apply the proposed method in more extensive practice.

1) As mentioned at the end of Section 5, studies about the storage degradation simulation methods for more key parts of other complex EMCs will be carried out by the authors in future.
2) The virtual testing method for simulating complex and comprehensive effects of multiple environmental stresses (for example, temperature stress is accompanied by intermittent electrical and vibration stress) under generalized storage conditions, which is another future work of the authors.

## Author contribution statement

Yigang lin: Conceived and designed the experiments; Analyzed and interpreted the data; Contributed reagents, materials, analysis tools or data; Wrote the paper. Tao He: Performed the experiments; Contributed reagents, materials, analysis tools or data. Haotong Zhu: Analyzed and interpreted the data. Yichen Lin: Performed the experiments; Analyzed and interpreted the data. Qiuying Chen: Analyzed and interpreted the data; Contributed reagents, materials, analysis tools or data; Wrote the paper.

## Data availability statement

Data will be made available on request.

## Funding

This research was funded by the 10.13039/501100004731Zhejiang Provincial Natural Science Foundation of China [grant number LY23E070001] and Key Science and Technology Research Project of Wenzhou of China [grant number ZG2022002 and ZG2021026].

## Declaration of competing interest

We declare that we have no financial and personal relationships with other people or organizations that can inappropriately influence our work, there is no professional or other personal interest of any nature or kind in any product, service and/or company that could be construed as influencing the position presented in, or the review of, the manuscript entitled.
